# A very versatile molecular machine

**DOI:** 10.1128/jb.00351-24

**Published:** 2024-09-30

**Authors:** Michael D. Manson

**Affiliations:** 1 Department of Biology, Texas A&M University, College Station, Texas, USA; NCBI, NLM, National Institutes of Health, Bethesda, Maryland, USA

**Keywords:** molecular motors, 5:2 symmetry, rotation

## Abstract

In this issue (J Bacteriol. 206: e0014024, https://doi.org/10.1128/jb.00140-24), Ridone and Baker describe hybrids between two 5:2 heteroheptameric ion-powered motors. Chimeras were constructed between stator units of a bacterial flagellum and ExbBD of the Ton outer-membrane transport system. Only one of the 14 hybrids supported swimming in *Escherichia coli*. Three additional residue changes at sites distant from the hybrid region enhanced motility. This work suggests that flagellar stator units and ExbBD share an ancestor that diverged during evolution to perform different tasks.

## COMMENTARY

Man-made motors contain a few basic components. There are wheels, shafts, gears, screws, pistons, cams, and a few other basic parts that can be combined in different ways to make a wide variety of machines that carry out diverse functions. Humans began using shafts and wheels about 10,000 years ago with the advent of agriculture. Screws and gears came somewhat later. Pistons and cams had to wait until the 19th-century AD.

Nature was far ahead of us in innovation, but we were slow to understand. Knowledge of rotation in biology came in 1973 when Howard Berg proposed that bacteria swim by rotating helical, screw-like flagellar filaments (1). It has become increasingly clear over the past 50 years that flagella and other biological machines are made up of a few basic parts.

Bacterial flagella presumably evolved several billion years ago, but they were probably not the first rotary biological machines. That honor falls to the F_O_F_1_ ATP synthase/ATPase (2, 3). It evolved in bacteria, but it is also present in a “borrowed” form in bacteria-derived organelles: mitochondria in virtually all eukaryotic cells and chloroplasts in algae and plants.

For a long time, the bacterial flagellum seemed to be a unique but widely distributed feature across almost all taxonomic groups of bacteria. The flagellum was initially described as consisting of two basic components: a basal body-hook-filament complex that extends out of the cell into the medium and “stator” complexes embedded in the cytoplasmic membrane and firmly anchored to the rigid and massive bacterial cell wall. The stator complexes generate torque to spin the flagellum. It was shown that stator units can freely associate and dissociate with the basal body (4) and that they use a transmembrane ion motive force to conduct a flow of ions into the cell to provide the energy for flagellar rotation (5, 6).

There our understanding stood for 40 years. The bacterial flagellum was used by Michael Behe in his 1996 book *Darwin’s Black Box* (7) as an example of irreducible complexity that required intelligent design and could not have evolved. However, his argument is flawed. Different elements of the bacterial flagellum exist in structures that perform other functions in the cell, although it is still not clear exactly how they were recruited to form a flagellum.

Then came 2020, which brought us not only COVID-19 but also startling insight into how an ion current can be converted into torque. Two research groups working independently to resolve the structure of the stator through high-resolution cryoelectron microscopy (cryoEM) came to the same conclusion (8, 9). The stator units are not, as had been thought, composed of four A subunits and two B subunits. They have five A subunits and two B subunits.

That extra A subunit may not seem like a huge discovery, but to those who seek to explain flagellar rotation, it was eye-opening. The stator units were not, as had been envisioned, pushing on the C-ring of the rotor like parents standing around a merry-go-round for children. The subunit asymmetry of the stator suggested that the five A subunits rotate around the two central B subunits, which are firmly attached to the cell wall. The rotating rings of A subunits can then interact with the C-ring of the basal body like multiple gears to drive the flagellar rotation. The complexes serve as stators because they are anchored to the cell wall. Changes in the orientation of the FliG protein in the C-ring of the basal body can explain how rotation of the stator A rings in the clockwise direction can drive both counterclockwise and clockwise rotation of the flagellum (10). A simple and elegant model of how this might be accomplished is presented in Figure 6 of that paper.

No one has directly visualized the rotation of the A subunit rings of the stator units. However, the idea is so attractive that it immediately caught on with the group of scientists who study bacterial motility. An important corollary realization was that machines that drive different energy-coupled functions at the cell surface are powered by similar 5:2 motors. This list includes the ExbBD units of the Ton transport systems across the outer membrane (11), the energy-generating components of the Tol system involved in cell division (12), the Gld system involved in gliding motility (13), and the Zorya phage defense system (14).

A very strong inference drawn from the extreme diversity of functions taken on by heteroheptameric 5:2 motors is that there was an early common ancestor that was then repurposed by evolution to accomplish different tasks. It is uncertain what this common ancestor may have been. However, considerations of parsimony suggest that the 5:2 stator may have originally evolved as the ExbBD Tol system involved in cell division, a function that is primary and necessary in all prokaryotic cells (15).

To explore the evolutionary relationships among these different heptaheteromeric motors, Ridone and Baker (16) set themselves the ambitious goal of generating hybrid motors. They chose the sodium-ion-driven PomAPotB system, a hybrid between the hydrogen-ion-driven MotAB motor of *Escherichia coli* and the sodium-ion-driven PomAB motor of *Vibrio alginolyticus*, as the basic chassis. They then carefully chose stretches of PomAPotB residues to replace with equivalent residues from the ExbBD motor of the Ton system of *E. coli*.

They constructed 4 different hybrids of PomA and ExbB and 11 hybrids of PotB and ExbD. Only one of the 14 hybrids was functional, the one with the shortest length of ExbD. Seventeen residues in the ion-channel component of ExbD replace 17 residues in the ion-channel component of PotD. Three additional residue substitutions, two in PomA and one in PotD, are required to provide good motility.

It is fair to say that this work is surprising, but that could be for either of two diametrically opposed reasons. Some, like me, might be surprised that it was so difficult to find a functional chimera. My laboratory generated a functional hybrid between the homodimeric transmembrane nitrate/nitrite sensor NarX and the homodimeric Tar chemoreceptor that functioned as a nitrate/nitrite repellent chemoreceptor for nitrate and nitrate (17). NarX and Tar are modular proteins with sensing and signaling domains, and choosing a convenient splice site between the domains was straightforward. Additional mutations could be introduced into the sensing domain of NarX that changed the hybrid protein into an attractant sensor for nitrate (18). So, making hybrids between evolutionarily related proteins and then tuning them with additional mutations to carry out different functions should be easy. Right?

An opposing view is held by Richard Berry, who knows far more about motors with 5:2 symmetry than I do (19). He finds it remarkable that any functional hybrid between two complex heptaheteromeric macromolecular motors could be generated. If they had a common ancestor, it existed long ago, and flagellar stators and ExbBD took separate evolutionary paths to carry out very different tasks. The stator units must interact with the bacterial cell wall and with the C-ring in the rotor of the basal body. They also have to transition between floating free in the membrane with their ion channels closed and associating with the C-ring concomitantly with binding strongly to the cell wall and unplugging their ion channels. ExbBD must interact with TonB to transduce energy to gated pores in the outer membrane. In addition, the three-dimensional structure of the 5:2 heterohexamer is far more complex than that of the extended homodimeric chemoreceptor and transmembrane sensor kinases.

Another point of interest is that the PomAPotB stator uses a current of sodium ions to drive flagellar rotation. ExbBD is thought to use a proton motive force as a source of energy. The hybrid PomAPotD stator, in which most of the ion-channel component of PotD derives from ExbD, uses sodium ions to power motility. This result suggests that sodium-ion-driven and hydrogen-ion-driven 5:2 motors have very similar ion channels. It is noteworthy that the sodium ion and the hydronium ion (H_3_O^+^) have nearly equal dimensions. The ability to exchange components of the ion channels of sodium-ion and hydrogen-ion motors with 5:2 symmetry suggests that the critical Asp residue in the channel, thought to be required for binding a sodium ion or for protonation, may actually bind a hydronium ion rather than become protonated. To test this idea, high-resolution cryoEM might be used to image the oxygen of a water molecule bound to the Asp residue of an ExbBD or MotAB ion channel. The ability to detect water molecules and the Na^+^ ion bound to the Asp24 residue in the 2.33 Å cryoEM structure of PomAB (20) suggests that this should now be technically feasible.

In summary, four important lessons can be drawn from this work. First, it provides support for the idea that flagellar stators and the ExbBD system share a common ancestor. Second, the rarity of functional chimeras and the fact that additional mutations are necessary to establish reasonably efficient function emphasize how finely tuned these different systems must be to interact with different cellular components to carry out disparate functions. Third, it shows that the creation of chimeras is a viable way to approach the function of seemingly related but evolutionarily distant macromolecular complexes. Finally, it proves that fortune favors the brave. Science advances when investigators are willing to take on high-risk endeavors to answer important questions.
